# Supplementary material for: Concept of an artificial muscle design on polypyrrole nanofiber scaffolds
Source: PLoS One. 2020 May 11;15(5):e0232851. doi: 10.1371/journal.pone.0232851 (PMC7213722; doi:10.1371/journal.pone.0232851)
Supplement: S1 Fig — (DOCX) [file pone.0232851.s001.docx]

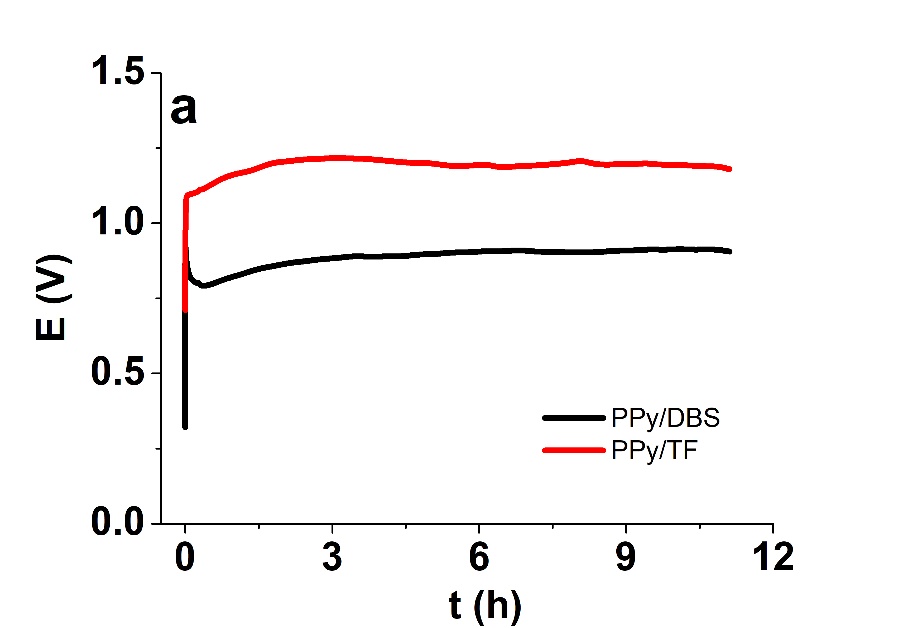


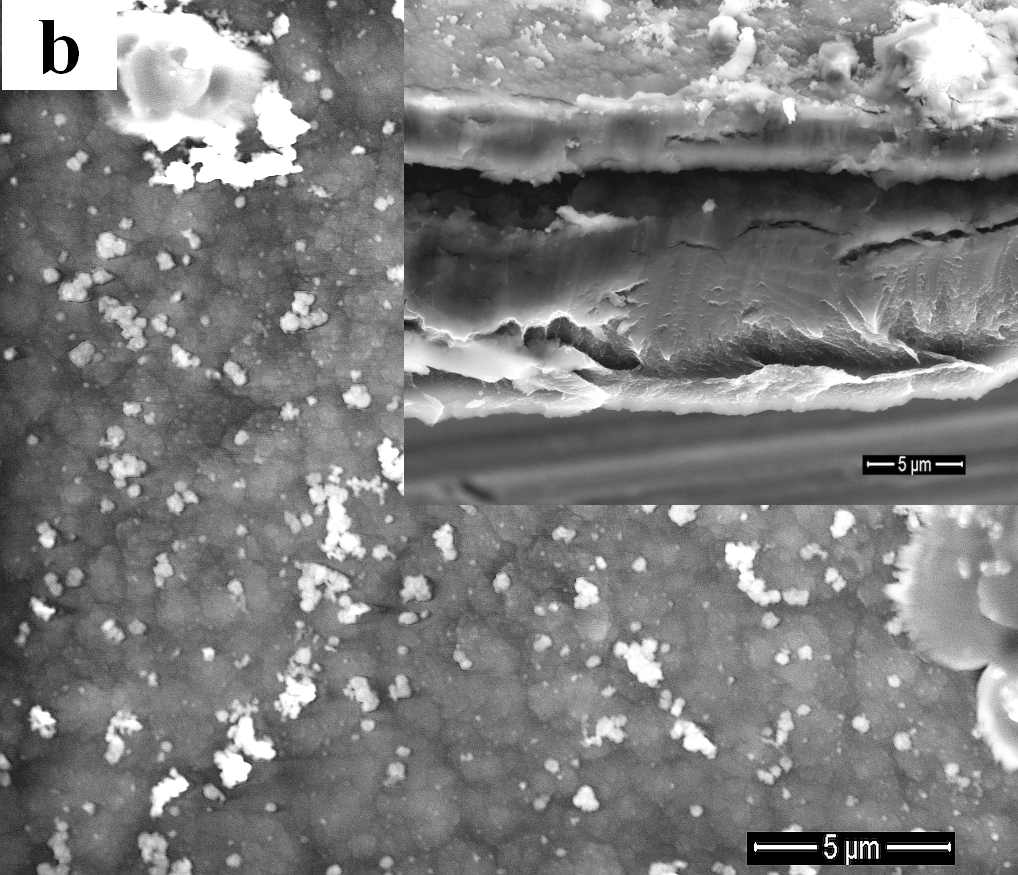

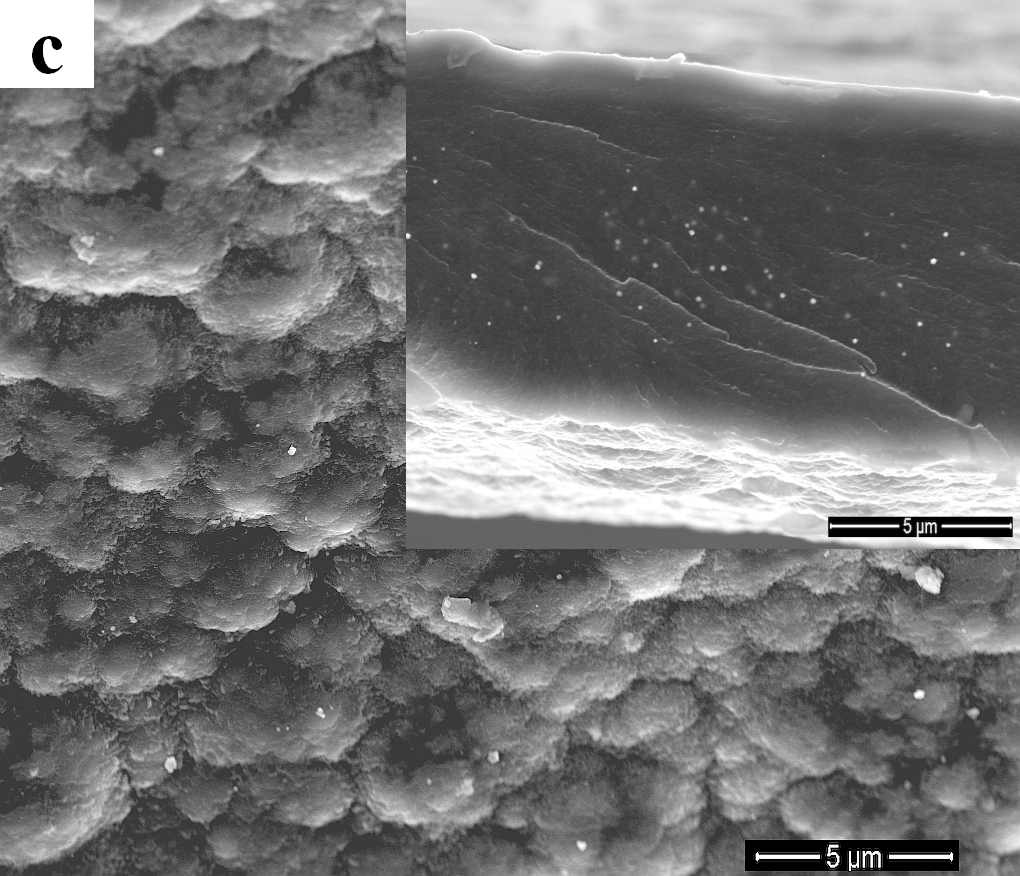


Figure S1. a: Galvanostatic (0.1 mA cm^-2^, 11.1 h (40,000 s), -40^0^C) electropolymerization in two electrode cell of PPy/DBS (black line) in 0.1 M NaDBS, 0.1 M Py in EG:Milli-Q (1:1) and PPy/TF (red line) in 0.1 M TBACF_3_SO_3_ and 0.1 M Py in propylene carbonate. The SEM surface images (scale bar 5 µm) with inset of cross section (scale bar 5 µm) are shown in b: for PPy/DBS and c: PPy/TF samples.
