# Supplementary material for: Concept of an artificial muscle design on polypyrrole nanofiber scaffolds
Source: PLoS One. 2020 May 11;15(5):e0232851. doi: 10.1371/journal.pone.0232851 (PMC7213722; doi:10.1371/journal.pone.0232851)
Supplement: S2 Fig — (DOCX) [file pone.0232851.s002.docx]

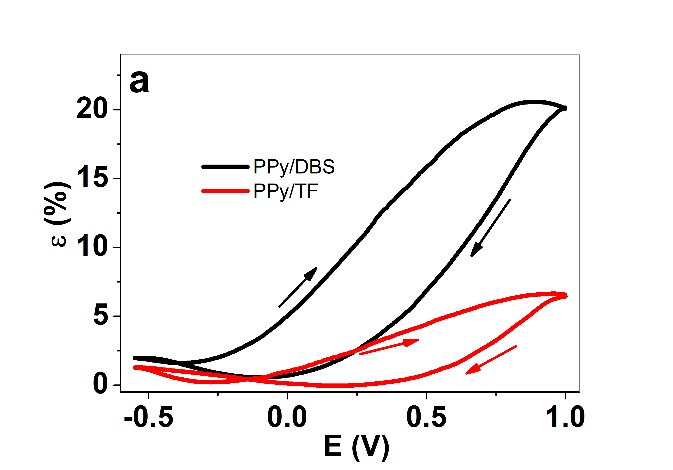

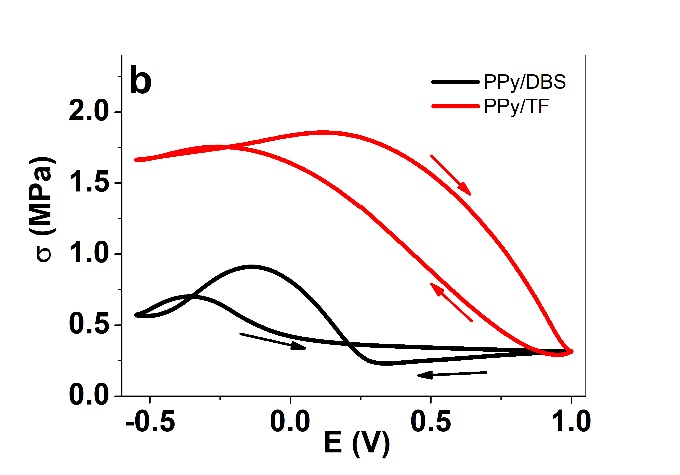

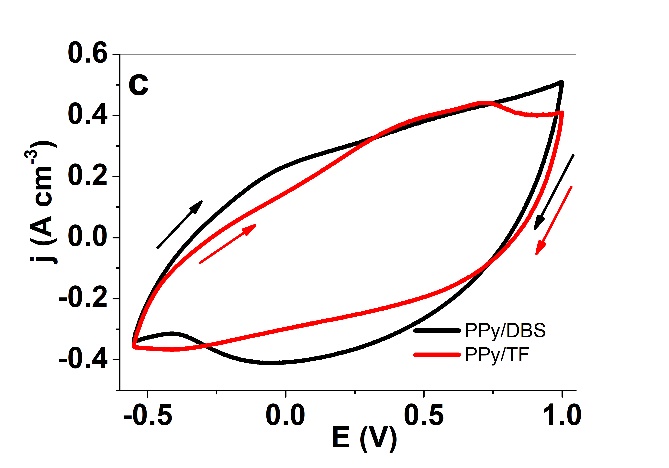

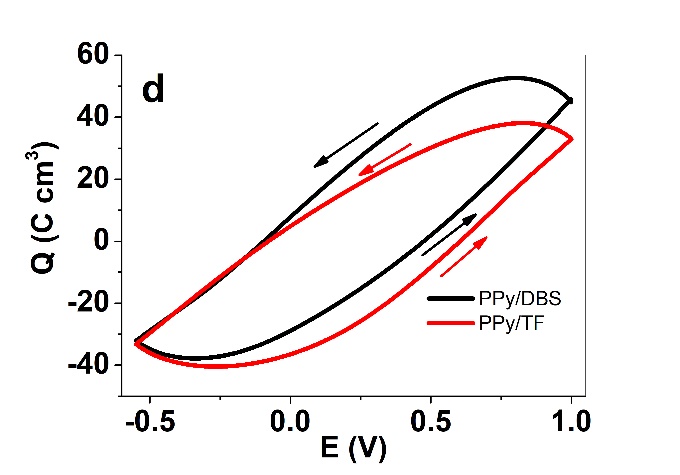


Figure S2. Cyclic voltammetry (scan rate 5 mV s^-1^) of free standing films of PPy/DBS (black line) and PPy/TF (red line) in LiTFSI-PC (potential range 1 V to -0.55 V against Ag/AgCl (3 M KCl) reference electrode, 4^th^ cycle) showing in a: strain ε; b: stress σ; c: current density j and d: charge density Q against potential E. The arrows indicate the start and ending of the cycle.
