# Supplementary material for: Concept of an artificial muscle design on polypyrrole nanofiber scaffolds
Source: PLoS One. 2020 May 11;15(5):e0232851. doi: 10.1371/journal.pone.0232851 (PMC7213722; doi:10.1371/journal.pone.0232851)
Supplement: S3 Fig — (DOCX) [file pone.0232851.s003.docx]

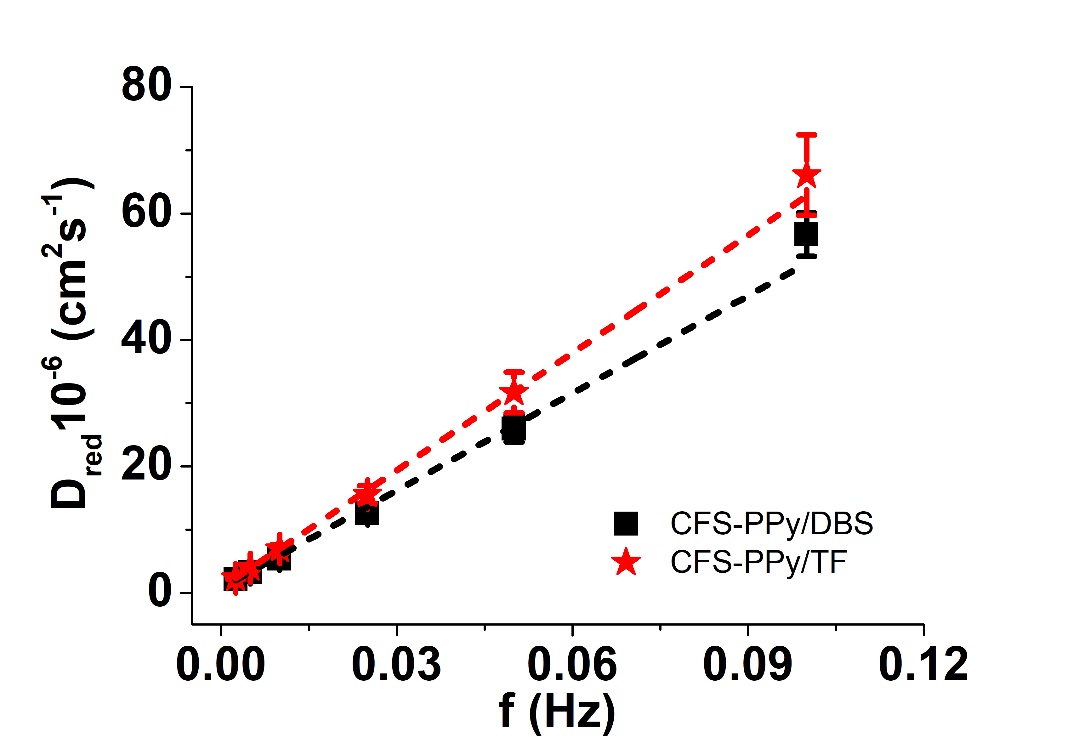


Figure S3. CFS-PPy/DBS (■) and CFS-PPy/TF (★) showing Diffusion coefficients D_red_ (at reduction) obtained from equations 2 and 3 against applied frequencies f. The dashed lines represent the linear fit and shown here for orientation only.
