# Supplementary material for: Concept of an artificial muscle design on polypyrrole nanofiber scaffolds
Source: PLoS One. 2020 May 11;15(5):e0232851. doi: 10.1371/journal.pone.0232851 (PMC7213722; doi:10.1371/journal.pone.0232851)
Supplement: S1 Table — (DOCX) [file pone.0232851.s004.docx]

Table S1. CFS-PPy/DBS and CFS-PPy/TF samples (more than three for each samples) in mean values with standard deviation at different frequencies f of 0.0025 Hz to 0.1 Hz of charge densities Q_ox_ and strain ε

| f [Hz] | CFS-PPy/DBS | | CFS-PPy/TF | |
| --- | --- | --- | --- | --- |
|  | ε [%] | Q_ox_ [C cm^-3^] | ε [%] | Q_ox_ [C cm^-3^] |
| 0.0025 | 21.2 ± 1.84 | 29.7 ± 2.12 | 10.1 ± 0.86 | 34.1 ± 2.87 |
| 0.005 | 17.1 ± 1.15 | 20.7 ± 1.72 | 8.3 ± 0.62 | 23.4 ± 2.12 |
| 0.01 | 12.4 ± 1.30 | 11.3 ± 1.03 | 6.3 ± 0.54 | 13.8 ± 1.26 |
| 0.025 | 6.1 ± 0.60 | 3.4 ± 0.32 | 3.2 ± 0.25 | 5.0 ± 0.43 |
| 0.05 | 3.6 ± 0.26 | 1.3 ± 0.11 | 1.7 ± 0.15 | 1.9 ± 0.13 |
| 0.1 | 1.8 ± 0.13 | 0.7 ± 0.06 | 1.1 ± 0.11 | 0.7 ± 0.05 |
