# Supplementary material for: Concept of an artificial muscle design on polypyrrole nanofiber scaffolds
Source: PLoS One. 2020 May 11;15(5):e0232851. doi: 10.1371/journal.pone.0232851 (PMC7213722; doi:10.1371/journal.pone.0232851)
Supplement: S2 Table — (DOCX) [file pone.0232851.s005.docx]

Table S2. CFS-PPy/DBS and CFS-PPy/TF samples (more than three for each samples) in mean values with standard deviation at different frequencies f of 0.0025 Hz to 0.1 Hz showing diffusion coefficients D_ox_ (D_red_) and strain rates ν_ox_

| f [Hz] | CFS-PPy/DBS | | CFS-PPy/TF | |
| --- | --- | --- | --- | --- |
|  | D_ox_ (D_red_)  10^-6^ [cm^2^ s^-1^] | ν_ox_ [% s^-1^] | D_ox_ (D_red_)  10^-6^ [cm^2^ s^-1^] | ν_ox_ [% s^-1^] |
| 0.0025 | 2.09 ± 0.15  (2.15 ± 0.18) | 0.21 ± 0.02 | 2.43 ± 0.22  (2.32 ± 0.21) | 0.023 ± 0.002 |
| 0.005 | 2.95 ± 0.25  (3.23 ± 0.32) | 0.23 ± 0.02 | 3.73 ± 0.34  (3.96 ± 0.35) | 0.043 ± 0.004 |
| 0.01 | 5.0 ± 0.49  (5.4 ± 0.52) | 0.25 ± 0.02 | 6.44 ± 0.60  (6.92 ± 0.67) | 0.083 ± 0.008 |
| 0.025 | 11.80 ± 0.98  (12.54 ± 1.25) | 0.31 ± 0.03 | 14.89 ± 1.27  (15.56 ± 1.39) | 0.14 ± 0.011 |
| 0.05 | 24.51 ± 2.28  (25.95 ± 2.12) | 0.42 ± 0.04 | 30.67 ± 2.94  (31.68 ± 3.23) | 0.23 ± 0.021 |
| 0.1 | 50.08 ± 5.50  (56.65 ± 3.46) | 0.66 ± 0.06 | 65.22 ± 3.06  (66.07 ± 6.37) | 0.45 ± 0.040 |
