# Supplementary material for: Concept of an artificial muscle design on polypyrrole nanofiber scaffolds
Source: PLoS One. 2020 May 11;15(5):e0232851. doi: 10.1371/journal.pone.0232851 (PMC7213722; doi:10.1371/journal.pone.0232851)
Supplement: S3 Table — (DOCX) [file pone.0232851.s006.docx]

Table S3. CFS-PPy/DBS and CFS-PPy/TF samples (more than three for each samples) in mean values with standard deviation at frequency 0.1 Hz presenting strain ε at cycle numbers.

| Cycle number | CFS-PPy/DBS, strain ε [%] | CFS-PPy/TF, strain ε [%] |
| --- | --- | --- |
| 50 | 1.84 ± 0.13 | 1.10 ± 0.12 |
| 200 | 1.83 ± 0.14 | 1.05 ± 0.10 |
| 400 | 1.80 ± 0.11 | 1.03 ± 0.10 |
| 600 | 1.78 ± 0.12 | 0.99 ± 0.08 |
| 800 | 1.76 ± 0.16 | 0.98 ± 0.09 |
| 1000 | 1.75 ± 0.15 | 0.95 ± 0.09 |
